# Effect of negative valence on assessment of self-relevance in female patients with borderline personality disorder

**DOI:** 10.1371/journal.pone.0209989

**Published:** 2019-01-10

**Authors:** Pegah Sarkheil, Niko Goik, Camellia N. Ibrahim, Frank Schneider

**Affiliations:** 1 Department of Psychiatry, Psychotherapy and Psychosomatics, Medical school, RWTH Aachen University, Aachen, Germany; 2 JARA Institute Brain Structure Function Relationship, Research Center Jülich and RWTH Aachen University, Aachen, Germany; 3 Department of Cognitive Neuroscience, Faculty of Psychology and Neuroscience, Maastricht University, Maastricht, The Netherlands; 4 University Hospital Düsseldorf, Düsseldorf, Germany; University of Bern, SWITZERLAND

## Abstract

**Background:**

A disturbed self-image is central to the characteristic symptoms of borderline personality disorder (BPD). Evaluations of self-relevance (SR) are highly important in cognitive and emotional processing of information and adaptive behavior.

**Method:**

In the current study, we used affective statements to investigate if SR is altered in patients with higher scores on Borderline Symptom List (BSL-95). Forthyfemale adults with BPD and 20 healthy participants assessed a set of stimuli consisting of sentences in third-person for relevance to self.

**Results:**

BPD patients exhibited a higher SR for negative contents as compared to healthy controls (p < .001). Furthermore, a significant positive correlation coefficient was found between the increased bias in evaluating the SR of stimuli and borderline symptom severity scores, as measured by BSL-95 questionnaire (r = 0.67, p < .001). This effect persisted after controlling for depressive symptoms by a partial correlation analysis.

**Conclusion:**

Our results revealed an enhanced SR for negative statements, which was related to the severity of individuals’ BPD symptoms. These findings add to the diagnostic information regarding the disturbed organization of self in this clinical population. We suggest the maladaptive evaluation of SR offers an important treatment target for therapeutic approaches to BPD.

## Introduction

One important aspect of self is evaluating relevance of environmental and mental events to self. Self-relevance (SR) is defined as top-down mechanism [1] with an impact on memory, perception, decision making and goal-directed behavior [2]. In the brain, a large-scale supramodal system has been proposed by neuroimaging studies to mediate the appraisal of self-relevant content, which includes the cortical midline structures and areas in the ventromedial prefrontal cortex [1,3,4] as well as several other regions including insula, basal ganglia and amygdala [5].

Clinical observations have reported aberrant judgements of SR in psychopathological conditions. Attribution of negative affects to the self has been acknowledged to be a characteristic of depressive syndromes [6] and the attainment of more positive interpretations for self has been posited as a desirable goal in clinical treatments of dysphoric individuals [7]. A dysfunctional differentiation between self-relevant and -irrelevant information has been suggested to underlie positive symptoms of schizophrenia thorough aberrant attribution of salience [8].

BPD is a severely impairing mental disorder, which affects 2–5% of adult population [9]. Individuals with this disorder suffer from affective dysregulation, heightened impulsivity, an unstable self and difficulties in interpersonal functioning [10], leading to a limited quality of life for the afflicted individuals and serious impact on mental health services. Impairments of the self is considered to be a central area of difficulty in borderline personality disorder (BPD) [11,12], which highlights the relevance of SR investigations to our understanding of BPD psychopathology.

In fact, modulatory role of SR evaluations on cognitive and emotional processing of information and especially on attention-emotion interactions in individuals with BPD has been already proposed [13], which is expected to have an influence on the goal-directed behavior. Based on a meta-analysis of attention bias studies in BPD, personally relevant negative words drive stronger attentional biases, as compared to the other negative material [14]. Recent evidence suggests that in individuals with BPD, the relevance to self can generate negative biases in information processing, which might enhance the disapproving view of themselves [15]. An alternated electrocortical reactivity has also been shown during processing of negative self-relevant information in females with BPD [16].

Altogether, this evidence suggests a modulatory role of SR evaluations on cognitive and emotional processing of information, which is critical in ruminations and dysfunctional behaviours in BPD. An increased tendency to attribute events to themselves have been shown in BPD [17]. To date, however, there has been limited empirical research investigating the judgments of SR in this clinical population. Especially the interaction of maladaptive attention and evaluation bias toward negative affects [18–21] and the increased intensity of negative emotions [22] in BPD has not been investigated yet.

In the present study, we relied on a challenge method by presenting written affective statements, which are phrased in third-person and attempted to probe if valence of the stimuli has an effect on the attribution of SR in BPD and control groups. The study assessed two hypotheses about BPD patient population. First, it was hypothesized that, negative sentences would trigger a higher SR. An interconnection between the appraisals of affect and SR is expected based on the widely recognized Linehan’s model for BPD [23] that suggests the emotional dysregulation to be responsible for formation of a disturbed self and other features of BPD. IQ and education was also based on the Linehan’s theory, supposing deficits of appropriate emotion regulation strategies to underlie maladaptive cognitions and behaviours [24]. Among the common emotion regulation strategies, cognitive reappraisal (changing the way one thinks about potentially emotion-eliciting events) is associated with healthier patterns of affect-related functioning as compared to expressive suppression (changing the way one behaviorally responds to emotion-eliciting events) [25]. We therefore expected a correlation between deficiencies in cognitive reappraisal and SR processing in BPD. The third hypothesis was that the severity of disorder-related symptoms would be related to the strength of the maladaptive processing of SR. Given the convoluted structure of the BPD symptoms, disturbances of the self are expected to interrelate with other difficulties of the BPD patients.

To examine the above hypotheses and derive clinical applications from the results of the research questions, we chose an approach that merged an experimental behavioral paradigm and BPD symptom assessment based on standardized questionnaires. We first investigated whether a maladaptive SR can be detected in BPD by an assessment task, and then related the intensity of SR to the severity of the BPD symptoms and emotion regulation strategies in patients.

## Methods

### Participants

We studied 40 female BPD patients (mean age: 24.8 years ± 6.5 [range = 19–42]) and 20 female healthy volunteers (mean age: 26.9 years ± 4.6 [range = 20–39]) with no current or previous psychiatric disorders and psychotropic medication, which constituted our healthy control (HC) group. Because of the gender differences in behavioral presentation of BPD [26], only female subjects were included. Patients were recruited from inpatient psychiatric unit at RWTH University Hospital Aachen. All participants from the BPD group met the diagnostic criteria for Borderline Personality disorder according to the “International Statistical Classification of Diseases (ICD-10)” as assessed by a psychiatrist. The reliability of diagnoses was additionally ensured through frequent team meetings at the psychiatric unit for all patients. 22 out of 40 patients (55%) also had a comorbid psychiatric diagnosis, and 25 patients (62.5%) received psychotropic medication (see Table 1 for specific diagnoses and medication). The healthy volunteers were recruited through advertisement and matched regarding age and gender.

**Table 1:** Psychotropic medications and comorbidities in BPD group.

Psychotropic medication	n (%)
medication free	15(37.5)
SSRI (Fluoxetine/Escitalopram/Sertralin)	8(20)
Quetiapine	4(10)
Venlafaxine	3(7.5)
Mirtazapine	2(5)
SSRI+antipsychotics(Quetiapine/Aripiprazole)	2(5)
Aripiprazole+anticonvulsant(Lamotrigin/Topiromat)	2(5)
Bupropion	1(2.5)
Bupropion+Lamotrigin	1(2.5)
Venlafaxine+Quetiapine	1(2.5)
SSRI+Bupropion+Quetiapine	1(2.5)
**Psychiatric comorbidities**	
depression	14(35)
eating disorders	4(10)
depression+eating disorders	3(7.5)
depression+PTSD	1(2.5)

All participants were native German-speakers. Participants were excluded if they had experienced severe head trauma, acute psychotic symptoms, substance addiction or a history of alcohol or substance abuse within past 6 weeks. The patients with benzodiazepine medication were excluded. The study was approved by the local Ethics Committee (The Independent Ethics Committee, medical faculty, RWTH Aachen University (EK048-16)). All participants gave written informed consent and were compensated with 10€/h for their participation. Evaluations of the capacity to consent were performed as part of the screening by a board-certified psychiatrist.

### Questionnaires

Questionnaire data was obtained at the test day as follows: 1) All patients completed BSL-95 (Borderline Symptom List) [27] to assess borderline symptom severity. The BSL-95 is composed of 7 subscales and the items are rated by using a 5-step Likert scale (0 = not at all, 4 = very strong). The values of total scale and the subscales were calculated by use of the mean (sum of values of the items / number of valid items). Healthy participants completed BSL-23 (the shorter version of Borderline Symptom List) [28] as a method to screen for borderline-related symptoms while relieving them from the detailed BSL-95 version. 2) The German Version of [29] ERQ (Emotion Regulation Questionnaire) [30] was applied to all participants to assess two different emotion regulation styles: expressive suppression and cognitive reappraisal. The ERQ uses a 7-point scale (1 = strongly disagree to 7 = strongly agree). 3) All participants completed the German version of [31] BDI-II (Beck Depression Inventory-II) [32] to measure depressive symptoms.

### Stimuli and task

Our means for assessing the SR of information was using the non-personal statements in form of sentences with third-person pronouns. We selected 30 negative, 30 positive and 30 neutral terms from German version of [33] “Affective Norms for English Words (ANEW)” [34], which provides a set of normative emotional ratings for a set of words. The selection of the words was based on affective valence categories (negative, positive, neutral). Other measured dimensions, arousal and dominance, were balanced across the categories. Relations to personal attitudes and situations were not controlled, however, given the sample size of the sentence material (n = 90), a major correlation of individual differences with the evaluative outcome was not expected. We used the selected ANEW words to build 90 brief sentences (30 for each valence category) containing a third-person female personal pronoun (she, her) without reference to a noun. The sentence building strategy was to make very simple 4–6 word sentences. The absence of a pronoun reference was used to examine the degree of self-reference. A sample of sentences in original German language and their English translation is presented as supplementary item (S1 Table).

The participants viewed 90 different sentences of the three affect categories (30 sentences for each condition) for 6 seconds and thereafter were asked to judge either (1) How strong was the feeling that the sentence was about them (rating SR of the sentence) or (2) How positive the sentence was (rating valence of the sentence). The order of the sentences and question types were randomized for each participant. We instructed the participants to rely on their spontaneous and subjective sense of self-relevance and not to get engaged in complex comparisons with the objective norms. They responded by pressing a key on keyboard to choose a response on a 7-step Likert scale. For SR judgements, the scale poles were anchored between 1 = lowest score and 7 = highest score. For valence judgments, the assignment of 1 and 7 poles to “highly negative” and “highly positive” were randomized across subjects to avoid systematized associations of valence and SR ratings. A blank screen followed after participants made their responses to separate the trials for 6 seconds. The response time was not collected; however, the trials were terminated after 7 seconds, if no key response was registered. To prevent fatigue, the participants were allowed 2 short breaks in fixed intervals. A practice session with 10 trials was conducted before the main experiment.

### Statistical analysis

A 3-way mixed model ANOVA with one between-subjects factor (group: BPD vs HC) and two within-subject factors (task: SR rating vs valence rating and valence: negative, neutral, positive) was calculated. For interpretation of interaction results, independent t-test were applied to examine the group differences in the valence rating and SR rating of affective sentences. Further, we analyzed Pearson correlation coefficients between the SR and the BSL-95 scores of the patients. Williams’ t-test for comparing two non-independent correlations was applied for statistical comparison of correlations. The analysis was performed by SPSS (IBM SPSS Statistics for Windows, Version 21.0. Armonk, NY: IBM Corp.).

## Results

### Questionnaire data

Data from BDI-II and ERQ questionnaires were obtained from all participants. All patients completed BSL-95 and all healthy participants completed BSL-23. Questionnaire data is summarized in Table 2.

**Table 2:** Means and standard deviations of the questionnaire scores in BPD and HC group.

	Questionnaires [range]			
	BSL-95/BSL-23 ^a^ [0–4]	ERQ-Reappraisal [1–7]	ERQ-Suppression [1–7]	BDI-II [0–63]
BPD	2.07(±0.79)	3.30(±1.49)	4.11(±1.57)	34.32(±13.49)
HC	0.16(±0.14)	4.95(±0.90)	2.92(±1.02)	3.25(±2.75)

^a^ Mean of total scale for BSL-95 in BPS and BSL-23 in HC group

Our patient sample scored in the BSL-95 subscales as follows: self-perception (1.71 ± 0.92), affect regulation (2.41 ± 0.0.83), self-destruction (2.40 ± 1.11), dysphoria (3.23 ± 0.52), loneliness (2.00 ± 0.86), intrusion (1.19 ± 0.78), hostility (1.64 ± 0.99). Total BSL-95 score was 2.07 ± 0.79.

There was a significant difference of ERQ scores between groups: The patients scored significantly lower in cognitive reappraisal (3.30 ± 1.49) than the healthy controls (4.94± 0.89; independent t-test: t(58) = -4.5, p < 0.001). The opposite pattern emerged for emotion suppression (BPD: 4.11 ± 1.57, HC: 2.92 ± 1.03; t(58) = 3.03, p<0.005).

BDI-II scores in the BPD group (mean ± SD: 34.32 ± 13.42) were significantly higher than in the HC group (3.25 ± 2.75; independent t-test: t(58) = 10.14, p < 0.001). Patients scored higher than cutoff threshold for depression in BDI-II. To rule out possible interactions between depression and task performance BDI-II score was included as covariate in further analyses.

### Measuring SR and valence rating

**Fig 1:**
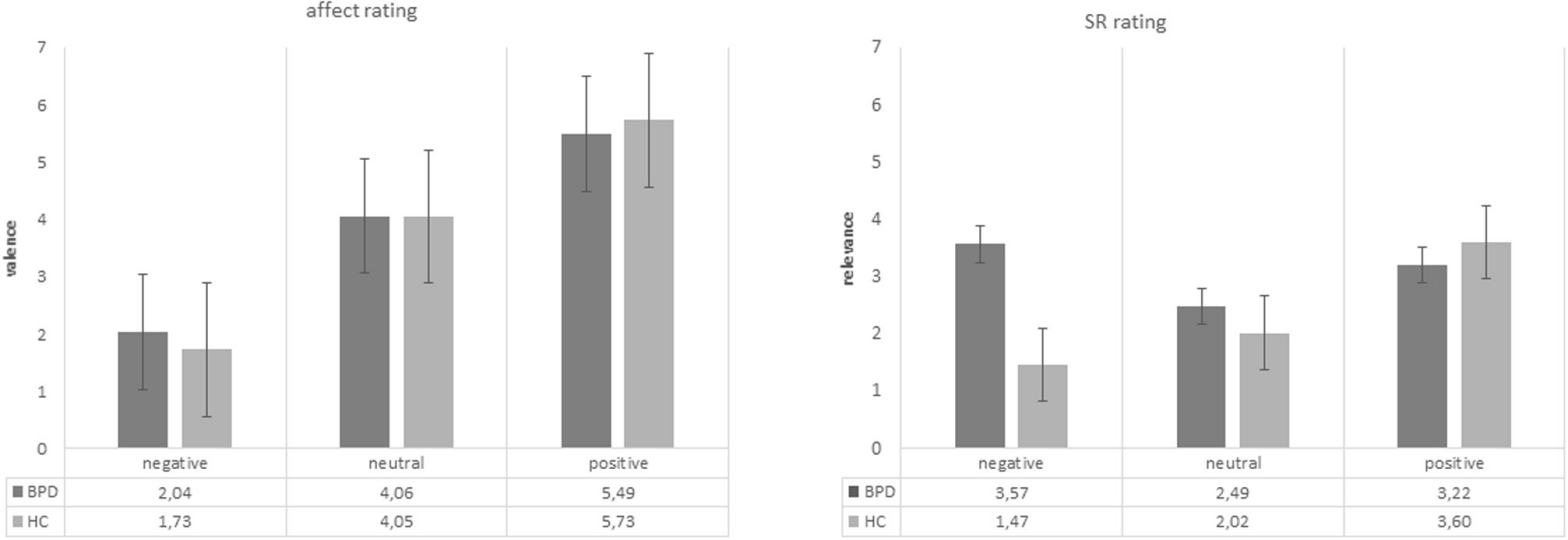
SR and valence rating. Ratings of SR (right) and affect (left) for patients (BPD) and controls (HC) as bar plots and value table is shown. Errorbars indicate the standard errors (SE). Y axis: 7-step Likert scale. A significant group difference was observed for SR of negative sentences.

The data were analyzed based on a 2 x 2 x 3 mixed model ANOVA, in which the between-subjects factor was group (BPD, HC), and the within-subjects factors were task (SR, affect rating) and valence (negative, neutral, positive). This analysis yielded a statistically significant group x task x valence interaction (F = 6.25, p = 0.004). Both group x task interaction and group x valence interactions were also significant (F = 5.92, P = 0.018; F = 18.18, P<0.001, respectively). To tease apart the specifics of the interactions we ran two-sample t-tests based on our hypothesis comparing the SR rating and affect rating of 3 affective sentence categories between the two groups (Fig 1). The t-test revealed that BPD and HC participants did not differ statistically in their affect rating of the negative, neural and positive sentences. The t-test, however, found that BPD group rated significantly higher SR for negative sentences (3.57 ± 1.21) compared to HC group (1.47 ± 0.31), t (58) = 6.5, p < 0.001. A significant group difference was not observed for SR of neutral and positive sentences.

### Correlations between SR rating and questionnaire scores

Correlation analyses focused on the significantly maladaptive SR in BPD group to investigate the role of the borderline symptom severity within the BPD group as measured by the questionnaires. Pearson correlation coefficients were computed between individuals’ SR and their BSL (total- and subscales), ERQ (CR and ES) and BDI-II scores. Table 3 presents the correlation statistics between SR and the questionnaire scores including the subscales.

**Table 3:** Pearson correlations (p values) between questionnaire scores and SR.

	SR of sentences			
		Negative	Neutral	Positive
BSL-95	Total score	.67** (<0.001)	.24 (0.14)	.10 (0.53)
	Self-perception	.39* (0.01)	.13 (0.42)	-.02 (0.93)
	Affect regulation	.68** (0<001)	.26 (0.11)	.11 (0.50)
	Self-destruction	.52** (<0.001)	.01 (0.95)	-.09 (0.58)
	Dysphoria	.24 (0.14)	-.18 (0.28)	-.28 (0.90)
	Lonliness	.65** (<0.001)	.30 (0.07)	.21 (0.20)
	Intrusion	.38* (0.02)	.36* (0.02)	.28 (.084)
	Hostility	.63** (0<001)	.21 (0.20)	.07 (0.67)
ERQ	Reappraisal	-.57** (<0.001)	-.07 (0.67)	.07 (0.67)
	Suppression	.09(0.60)	.12(0.50)	.04(0.80)
BDI_II	Total score	.43* (0.01)	.21 (0.25)	.06 (0.76)

**Fig 2:**
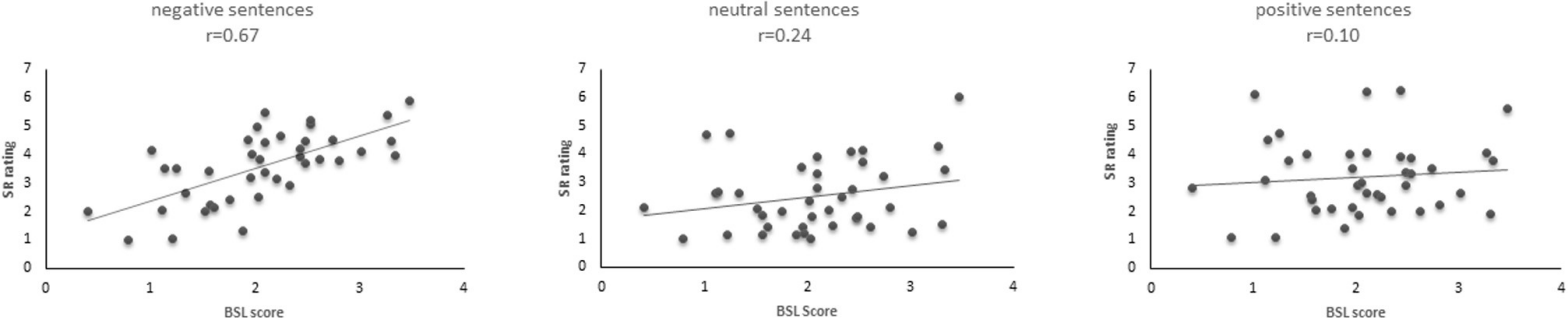
Correlations between SR ratings with BSL-95 scores. The correlation between SR ratings of negative sentences and BSL-95 scores was significant (r = 0.67).

As the main finding, a significant positive correlation coefficient was observed between the patients’ BSL-95 scores and SR of negative sentences (r = 0.67, p < 0.001), which was absent in case of neutral (r = 0.24, p = 0.14) and positive (r = 0.10, p = 0.53) sentences (Fig 2). The results of Williams’ t-test for comparing correlations indicated that the differences between these correlations were statistically significant (negative vs neutral (t = 3.87, p < 0.001) and negative vs positive (t = 4.72, P < 0.001)).

A partial correlation was run to determine the relationship between the patients’ BSL scores and SR, whilst controlling for BDI-II score. There was a positive partial correlation between BSL and SR of negative statements, while controlling for BDI-II score, which was statistically significant (r = 0.59, p < 0.001) and confirmed that depression did not have a significant influence in controlling for the relationship between BSL score and SR.

The correlation analysis yielded a significant negative correlation between the patients’ tendency to regulate their emotion using cognitive reappraisal and SR of negative sentences (r = -0.56, p < 0.001).

Multiple correlations have been tested between 7 main variables (BSL-95 score, ERQ-reappraisal score, ERQ-suppression score, BDI-II score, negative SR, neutral SR, positive SR) yielding 21 unique correlation tests. The above-mentioned significant correlations survived the Bonferroni adjusted significance level (0.05/21 = 0.002) for multiple tests.

## Discussion

To our knowledge, this represents the first study to explicitly tackle the judgements of SR in BPD by showing a deviancy in comparison to a healthy sample and, most importantly, a relationship between SR and BPD symptom severity. We designed an experimental paradigm to examine the experience of SR by exposing a variety of sentences containing a third-person personal pronoun to the participants and engaging them in an assessment task. The affect rating did not differentiate the groups. Both BPD and HC groups were able to distinguish the positive, negative and neutral categories of sentences in this paradigm. Our results revealed that, in assessing hypothetical statements, BPD patients excessively attribute negative statements to self. Within our BPD sample, patients with higher symptom severity showed an increased tendency to judge the negative contents as related to self. To examine if the SR deviation is unique to the BPD pathology or is driven by co-existing depression, we conducted a partial correlation analysis between BSL-95 and SR of negative sentences while controlling for BDI-II score. A positive correlation between SR and symptom severity emerged after controlling for BDI-II scores, verifying that this difference was not an artifact of depressive severity.

These findings contribute to understanding the link between the disturbed self and the clinical presentations of BPD. Most people endorse more positive features than negative features for the self, which promotes experiencing more favorable psychological and social outcomes [35]. In BPD, deviations from such self-enhancing tendencies are likely to affect the sense of well-being. Negative self-relevant cues are likely to contribute to a heightened instability of self-image and in this way further trigger downstream behavioral outcomes such as impulsive and maladaptive behavior. Although the results confirmed o Our second hypothesis our hypothesis regarding a bias toward the self-relevance of negative contents in BPD, it should be considered that some of it might be explained by the effect of other psychopathological changes, like disturbed mood, on evaluative judgements of patients.

One possible contributing factor to disturbances of self is an impact of emotional processes such as valence appraisals. Emotions guide us to navigate in our complex environment. In pathological states, however, emotions may lead to maladaptive cognitive functions, as illustrated for example by exaggerated attentional bias to threat in anxiety [36], preferential memory for negative events in depression [37] and negative bias in discrimination of negative and neutral facial expressions in BPD [38,39]. Neuroimaging investigations have already suggested dysfunctionality in the neural circuitry responsible for evaluation of emotional salience and conflict control [40]. Our stimuli set, therefore, consisted of sentences of different valence categories. The negative valence augmented the attribution of relevance to self in BPD, indicating a role for emotional appraisals in self-evaluative processes in this clinical population. It is possible that the negative contents trigger SR through salience mechanisms. Another proposal is that the enhanced SR for negative contents, as measured by our paradigm, derives from emotion regulation deficits in the BPD sample. Support for this proposal comes from our data, which showed a correlation between SR bias and deficits of cognitive reappraisal strategies as measured by ERQ. This is in line with Linehan’s model for BPD [23], which postulates that an underlying vulnerability to emotional dysregulation is the basis for evolution of a disturbed self-image and interpersonal impairments of individuals affected by BPD.

Another principal finding that emerged from the correlation analysis was that the SR bias was higher in patients with more severe symptoms. A severely affected patient suffers from a biased SR far more than a mildly affected individual. The implication is that the SR bias is relevant to the BPD pathology and cannot be generalized to the unspecific characteristics of the clinical sample. Self-relevance biases along with other self-related pathologies might contribute to impairments of the self-concept in BPD [41]. We argue that a disruption of the SR evaluation system in BPD may be relevant to the troubled self-image and likewise other typical difficulties in BPD like affective stability and interpersonal problems in BPD patients.

Our findings about increased attribution of negative statements to self in BPD fits very well into the attribution style theory [42], which focuses on the causal interpretation of events regarding to the self or others by the individuals. A framework for understanding psychopathological deviations such as depressive mood [43] and persecutory delusions [44] has been already suggested based on the attribution style theory. In respect of BPD, an enhanced internal attributions for negative events has been already shown [45].

In sum, it appears that the construct of self-relevance and other self-evaluative processes in BPD deserves further attention. It might be quite elucidating to refer to a negative SR as a basis for maladaptive interrelations of emotion and self. We suggest that the investigation of the material encoded in a self-relevant manner may prove useful for the understanding and treatment of BPD. Cognitive interventions should focus on modification of a biased attribution of negative contents to self, using affective training material.

## Limitations

We would like to acknowledge several limitations of our study, which should be considered in the interpretation of the results. Only female subjects were included, which limits the generalizability of the findings. IQ and education were not included as between-group matching variables. Most of the BPD patients were medicated, which may have affected some group differences. Regarding the stimuli, the effect of individual biography and pre-exposures may be more relevant for participants with BPD and the possible confounds associated with endorsed self-relevant statements were not explicitly examined.

## Supporting information

S1 Table Samples of sentences used to cue self-relevance. Terms taken from ANEW are underlined.
(PDF)
