# Supplementary material for: Effect of negative valence on assessment of self-relevance in female patients with borderline personality disorder
Source: PLoS One. 2019 Jan 10;14(1):e0209989. doi: 10.1371/journal.pone.0209989 (PMC6328147; doi:10.1371/journal.pone.0209989)
Supplement: S1 Table — Terms taken from ANEW are underlined. (PDF) [file pone.0209989.s001.pdf]

S1 Table: samples of sentences used to cue self-relevance. Terms taken from ANEW are underlined.

Category	Original German	English Translation
**Negative**	Die Abscheu hat sie gepackt.	She was disgusted.
	Es wurde brutal gegen sie vorgegangen.	She was treated in a brutal way.
	Die Leute halten sie für illoyal.	People consider her as disloyal.
	Das Elend ist ihre Zukunft.	Misery is her future.
	Sie muss zu einer Beerdigung.	She should attend a funeral.
**Neutral**	Sie schaltet den Motor an.	She turns on the engine.
	Der Tisch steht in ihrem Zimmer.	The table is in her room.
	Sie sitzt auf dem Stuhl.	She sits on the stool.
	Ihre Sachen sind im Korb.	Her stuff is in the basket.
	Jemand nennt ihren Namen.	Somebody calls her name.
**Positive**	Sie ist voller Leidenschaft.	She is full of passion.
	Ihr schmeckt das Essen.	The food tastes good to her.
	Die Leute denken, sie ist im Vorteil.	People believe, she has an advantage.
	Sie macht sich gemuetlich.	She is getting cozy.
	Ihr Baby ist da.	Her baby is there.
